# Reintroducing testosterone in the *db/db* mouse partially restores normal glucose metabolism and insulin resistance in a leptin-independent manner

**DOI:** 10.1186/s12902-018-0266-y

**Published:** 2018-06-13

**Authors:** Koichi Yabiku, Keiko Nakamoto, Akihiro Tokushige

**Affiliations:** 1 Division of Endocrinology, Diabetes and Metabolism, Hematology, Rheumatology (Second Department of Internal Medicine), Graduate School of Medicine, University of the Ryukyus, 207 Uehara, Nishihara, Okinawa, 903-0215 Japan; 2 GenomIdea Incorporated, Okinawa, Japan; 3 Clinical Pharmacology and Therapeutics University of the Ryukyus School of Medicine, Okinawa, Japan

**Keywords:** Testosterone replacement, Leptin signal knockout, Aromatization, Impaired glucose tolerance, Fatty liver

## Abstract

**Background:**

Testosterone signals through the androgen receptor (AR) and AR knockout mice develop obesity, suggesting a functional association between AR and leptin signaling. Furthermore, physiological blood concentrations of testosterone have been found to inhibit the development of arteriosclerosis, obesity and diabetes. However, these findings have not been verified by testosterone replacement in animal models and whether or not testosterone acts directly by activating AR to enhance leptin signaling, or indirectly by its conversion into estrogen remains unclear. Therefore, we investigated the effect of exogenously supplemented testosterone on glucose and lipid metabolism.

**Methods:**

Four-week-old male leptin receptor-knockout *db/db* mice were used as controls for a model of obesity retaining low testosterone. Mice were divided into sham-operated, castrated, or castrated and testosterone-supplemented groups and fed a high-fat diet (HFD) for 2 weeks from 5 weeks of age. Testosterone concentrations, blood glucose, plasma insulin levels, and intraperitoneal glucose tolerance and insulin tolerance were measured. At 7 weeks, triglyceride and glycogen content were measured in the liver and muscle. Lipid accumulation in the liver and soleus muscle was determined by immunohistochemistry with Oil Red O. Statistical analyses were performed using the Student’s *t*-test or ANOVA where applicable.

**Results:**

Lower testosterone levels in *db/db* mice compared with wild type (WT) *db/+* mice were associated with glucose intolerance and fatty liver. Furthermore, castrated male *db/db* mice at 4 weeks of age progressively developed glucose intolerance accompanying a 15% increase in liver fat. Male mice fed a HFD had lower levels of testosterone compared with those fed a normal diet. We found that exogenous testosterone replacement injected subcutaneously into castrated male *db/db* mice alleviated the exacerbation of fatty liver and glucose intolerance, suggesting a leptin-independent mechanism. This mechanism is most likely mediated through gonadal axis suppression in this mouse model.

**Conclusions:**

In summary, testosterone may use a novel pathway to complement leptin signaling to regulate glucose and lipid metabolism, and thus offers a new therapeutic target to treat metabolic disorders.

**Electronic supplementary material:**

The online version of this article (10.1186/s12902-018-0266-y) contains supplementary material, which is available to authorized users.

## Background

In addition to maintaining gametes, testosterone is responsible for maintaining and elevating daily activity levels in adult men. For example, not all men adapt to a decrease in testosterone with aging. A rapid decrease in testosterone levels with aging has been reported in many cases, leading to a marked impairment of quality of life (QOL) in middle-aged to elderly men [1–3]. A decrease in blood testosterone levels was recently shown to increase the incidence of all-cause mortality [4], in addition to metabolic syndrome (MetS) [5–7], type 2 diabetes [8–10] associated with the progression of visceral obesity and enhancement of insulin resistance, osteoporosis-associated fracture [11–13], and progression of arteriosclerosis [14–16]. Although testosterone was previously considered to be an arteriosclerosis-promoting hormone because endogenous testosterone was shown to decrease HDL-cholesterol levels, the action of testosterone within its physiological blood concentration range was demonstrated to have an inhibitory effect on the development and progression of lifestyle-related diseases such as arteriosclerosis, obesity and diabetes [8, 17]. However, whether a low testosterone level may be a potent predictor of clinically-important coronary arterial disease has not been confirmed in previous case-controlled and longitudinal studies [18, 19].

The results of animal studies on exogenous testosterone are varied, with both positive and negative effects being observed. For example, it has been shown to exacerbate hypertension and induce renal failure in male SHR rats [20], whereas amelioration of erectile dysfunction has been reported in castrated animals [21–24]. Testosterone is known to act through the androgen receptor (AR). In AR knockout (KO) mice, the development of male-specific delayed-onset obesity has been reported, and the activation of AR enhanced leptin-induced STAT3 nuclear translocation and transcription of leptin target genes, in an in vitro system [25–27]. Therefore, the functional association between AR and leptin signaling has recently attracted attention. Although more detailed evidence is needed, AR is assumed to exhibit an anti-obesity effect by enhancing leptin signaling, which has been shown to activate sympathetic nerves.

Whether the effect of endogenous testosterone is directly caused by testosterone or its active form, dihydrotestosterone (DHT), through AR, or by estrogen (testosterone is converted to estrogen by aromatase), has not yet been elucidated [28, 29]. Qiu Y et al. demonstrated that administration of a physiological level of the non-aromatized androgen, DHT, to an arteriosclerosis model prepared by feeding a high-cholesterol diet to orchidectomized rabbits directly acted against arteriosclerosis [30]. Furthermore, studies using aromatase KO mice confirmed close associations with, first, the energy metabolism-related factor, leptin, and then with other factors such as PPARγ [31]. However, this has not yet been verified in animal studies by actual testosterone replacement.

In this study, we initially investigated testosterone and glucose metabolism in *db/db* mice relative to WT *db/+* controls and assessed a normal chow diet (NCD) and HFD. Changes in testosterone levels with aging were also investigated in each group of mice, as was the effect of diet (NCD or HFD). Subsequently, the influence of exogenously-supplemented testosterone on glucose and liver steatosis was investigated in *db/db* (castrated or sham-operated) mice. To confirm whether aromatisation is important for testosterone effects, we used its inhibitor, anastrozole. Conditions that elevated testosterone levels were simulated by keeping male and female mice in the same cage during the reproductive period to investigate how testosterone levels responded in male mice.

## Methods

### Animal experiments

Male and female *db/+* heterogeneous mice (10 weeks of age) were purchased from the Jackson Laboratory (Sacramento, CA) and housed in a temperature-controlled room (22–23 °C) with a 12 h light/dark cycle. All animal experiments were approved by the Institutional Animal Care Committee of the Faculty of Medicine at the University of the Ryukyus (No. 5115). Male *db/+* and *db/db* offspring were weaned at 4 weeks of age, divided into two groups at 5 weeks of age, and then fed a NCD or HFD for 2 weeks. To measure the blood testosterone level, tail vein blood was collected at 7 weeks of age. Blood samples were centrifuged, and the plasma testosterone concentrations were determined using an enzyme-linked immunosorbent assay (ELISA) kit (Endocrine Technologies, Inc. USA).

### Castration of mice

Four-week-old male *db/db* mice were castrated according to the following procedure. Under barbiturate anesthesia, an incision was made approximately 2 cm cranial to the penis, and an approximately 1 cm incision was made in the exposed abdominal muscle layer. The testes, epididymides and vas deferentia were pulled out of the body while avoiding injury to the intestines. For each testis, the vas deferens was ligated at two sites and cut between ligations. The testes were then resected and the wound sutured. Testosterone replacement was initiated in 5-week-old mice.

### Testosterone injection and measurement of plasma estradiol levels

Testosterone propionate (Wako, Osaka, Japan) was dissolved in sesame oil (Sigma-Aldrich, Tokyo, Japan) and its concentration was adjusted (20 μg/μl). Testosterone was injected subcutaneously into each group of castrated mice at 1, 10 or 100 μg/g body weight/2 days for 2 weeks from 5 weeks of age, and blood estradiol levels were measured at 7 weeks of age using an ELISA kit (Endocrine Technologies, Inc. USA).

### Analysis of fuel homeostasis

Sham-operated, castrated, and castrated and testosterone-supplemented groups (*n* = 8–14 per group) were fed HFD for 2 weeks from 5 weeks of age, and then intraperitoneal glucose tolerance (ipGTT) and insulin tolerance (ipITT) tests were performed. At 7 weeks of age, triglyceride (TG) content and glycogen content were also measured in the liver and muscle. The mice were fasted overnight and injected intraperitoneally with glucose (1 g/kg) for ipGTT testing. Mice fasted for 4 h were injected intraperitoneally with REGULAR human insulin (Humulin R, 1.2 U/kg, Eli Lilly, Indianapolis, USA) for ipITT testing. Blood glucose values were determined using a Medisafe glucometer (Terumo, Tokyo, Japan) and plasma insulin levels were quantified using an ELISA kit (Shibayagi, Gunma, Japan). Tissue TG content was measured using an enzymatic assay method reported previously [32, 33]. To measure glycogen content, pieces of the liver were isolated from 7-week-old mice in a 16 h fasted or ad libitum-fed state, and then homogenized in 3% (*w*/w) perchloric acid on ice. An aliquot of the homogenate was incubated for 2 h at 40 °C with amyloglucosidase. The resulting glucose residues were quantified using an enzymatic glucose kit (Sigma-Aldrich, Tokyo, Japan), and the glycogen content was expressed as milligram (mg) per gram (g) of wet liver.

### Histology and immunohistochemistry

To evaluate lipid accumulation in the liver and soleus muscle, frozen sections were stained with filtered Oil Red O (in isopropanol) and visualized with 0.1% lithium carbonate. Oil Red O-positive areas in the sections were measured as previously reported [33, 34]. To evaluate the pancreatic β cell mass, 4-μm sections were treated with guinea pig anti-porcine insulin (1:200). The sections were incubated with biotinylated secondary antibodies and the signals were visualized with 2,3′ diaminobenzidine (DAB). Images were captured using a ScanScope Digital Slide Scanner (Aperio, Vista, CA) and the β cell mass was estimated.

### Immunoblotting and real-time PCR

Immunoblotting was performed as described previously [35]. Regular human insulin (5 units/kg mouse body weight) was injected into each group after a 16 h fast, and the liver and soleus muscle were excised 30 min after injection. The excised organs were frozen in liquid nitrogen and stored prior to immunoblotting. To prepare the samples for immunoblotting, the tissues were lysed at 4 °C in NP-40 lysis buffer (1% NP-40, 50 mM Tris-HCl (pH 8.0), 150 mM NaCl) containing proteinase and phosphatase inhibitors (Sigma, Tokyo, Japan). Twenty micrograms (20 μg) of total protein from each sample were then separated by electrophoresis on 10% denaturing sodium dodecyl sulfate-polyacrylamide gels and transferred to nitrocellulose membranes (Millipore, Billerica, MA, USA). After blocking with Tris-buffered saline containing 0.05% Tween 20, the membranes were incubated with an anti-phosphoserine Akt (Ser473) antibody (Cell Signaling Technology, Beverly, MA, USA) (RRID: AB_39825) followed by a horseradish peroxidase-conjugated anti-rabbit IgG. Bound antibodies were detected using an enhanced chemiluminescence (ECL) system (Amersham, Little Chalfont, UK). The bound antibodies were then stripped from the membranes, which were reprobed with an anti-Akt antibody (Cell Signaling Technology, Beverly, MA, USA) (RRID: AB_329827) to reveal the location and intensity of the 60-kDa-labelled band. Scion Image software (US National Institutes of Health) was used for quantitative analysis of the blots.

Liver and muscle tissues samples for real-time PCR were preserved in RNAlater (Qiagen, Tokyo, Japan) prior to the isolation of total RNA using the RNeasy Lipid Tissue Mini Kit (Qiagen, Tokyo, Japan). The RNA samples were treated with DNase and reverse-transcribed into cDNA using Superscript II (Life Technologies, Foster City, CA). The cDNA samples were treated with RNase and used for real-time RT-PCR with SYBR Green PCR Master Mix (Applied Biosystems, Foster City, CA) in an ABI StepOnePlus Real-time PCR System as described previously [36, 37]. The expression of mRNA for each target gene was normalized relative to that of glyceraldehyde 3-phosphate dehydrogenase (*Gapdh*). The sense and antisense primers were 5′-TCTGGGTGGCAGTGGTCGGA-3′ and 5′-TGGCCAGAGGGACTTCCTGGT-3′, respectively, for *G6pc*, 5′-CGCAGGACGCGGAACCATGT-3′ and 5′-CATGCTGCCAGCTGAGGGCT-3′ for *Pck1*, 5′-TGTCGCAGGTGGAGAGCGACT-3′ and 5’-TCACAGGCACGGCGCACAAT-3′ for *Gck*, and 5′-TGTGTCCGTCGTGGATCTGA-3′ and 5′-TTGCTGTTGAAGTCGCAGGAG-3′, respectively, for *Gapdh*. The cycle number at which fluorescence exceeded the threshold of detection (CT) for *Gapdh* was subtracted from that of the target gene in each well (ΔCT). The percentage change in expression, relative to that of the vehicle-treated group, was defined as (2 − ΔΔCT × 100), where ΔΔCT = ΔCT for the intervention group − ΔCT for the vehicle-treated group.

### Continuous anastrozole infusion using an osmotic pump and subcutaneous implantation of a testosterone pellet in the neck

To block the conversion of testosterone to estradiol (aromatization), the aromatase inhibitor, anastrozole (AstraZeneca, London, UK), was dissolved in 0.3% hydroxypropylcellulose and 0.9% NaCl, added to an osmotic mini-pump (Alzet, Cupertino, CA), and delivered under the skin via an indwelling catheter in the back of 4-week-old castrated mice (the continuous infusion rate of anastrozole was adjusted to 200 μg/day). To adjust the blood testosterone levels to a steady state, a testosterone pellet (15 mg/tablet) (Nacalai Tesque, Kyoto, Japan) was simultaneously implanted under the neck skin of 4-week-old castrated mice using a precision trochar (a placebo pellet was implanted in control mice). This testosterone treatment produces maximal stimulation of spermatogenesis [38]. Glucose and lipid metabolism were similarly evaluated in 7-week-old mice, at which time the blood estradiol levels were also measured to confirm that they were below the level of sensitivity.

### Time-course evaluation of blood gonadotropin and testosterone levels and evaluation of testosterone using the hCG load test

Changes in blood testosterone and gonadotropin (luteinizing hormone (LH) and follicle stimulating hormone (FSH)) levels with aging were measured in male *db/db* and *db/+* mice from 4 weeks to 1 year of age and also according to the diet given (NCD and HFD) using an ELISA kit (CUSABIO, Wuhan, China). Ten-week-old male and female mice were kept in the same cage (separated by a fence to avoid copulation) for 2 weeks (male *db/db* mice × female *db/db* mice, male *db/db* × female *db/+*, male *db/+* × female *db/db*, and male *db/+* × female *db/+*), and blood testosterone levels were measured in the male mice. Control male mice were maintained without a female for 2 weeks from 10 weeks of age, and their testosterone levels were measured at 12 weeks of age. In addition, human chorionic gonadotropin (hCG) (Sigma, Tokyo, Japan) in PBS was administered (0.5 IU/g body weight) to 12-week-old mice, and increases in blood testosterone levels were compared between the groups.

### Statistical analysis

Data are presented as means ± s.e.m. Statistical analyses were performed using Student’s *t*-test or ANOVA with Tukey’s HSD multiple comparisons as appropriate. Differences were considered significant at *P* < 0.05. In addition, power calculations were performed based on average value differences and common standard deviations in each group, with the two-sided α-error set at 0.05.

## Results

### Influence of obesity and fatty foods on low circulating testosterone levels in mice

**Fig. 1:**
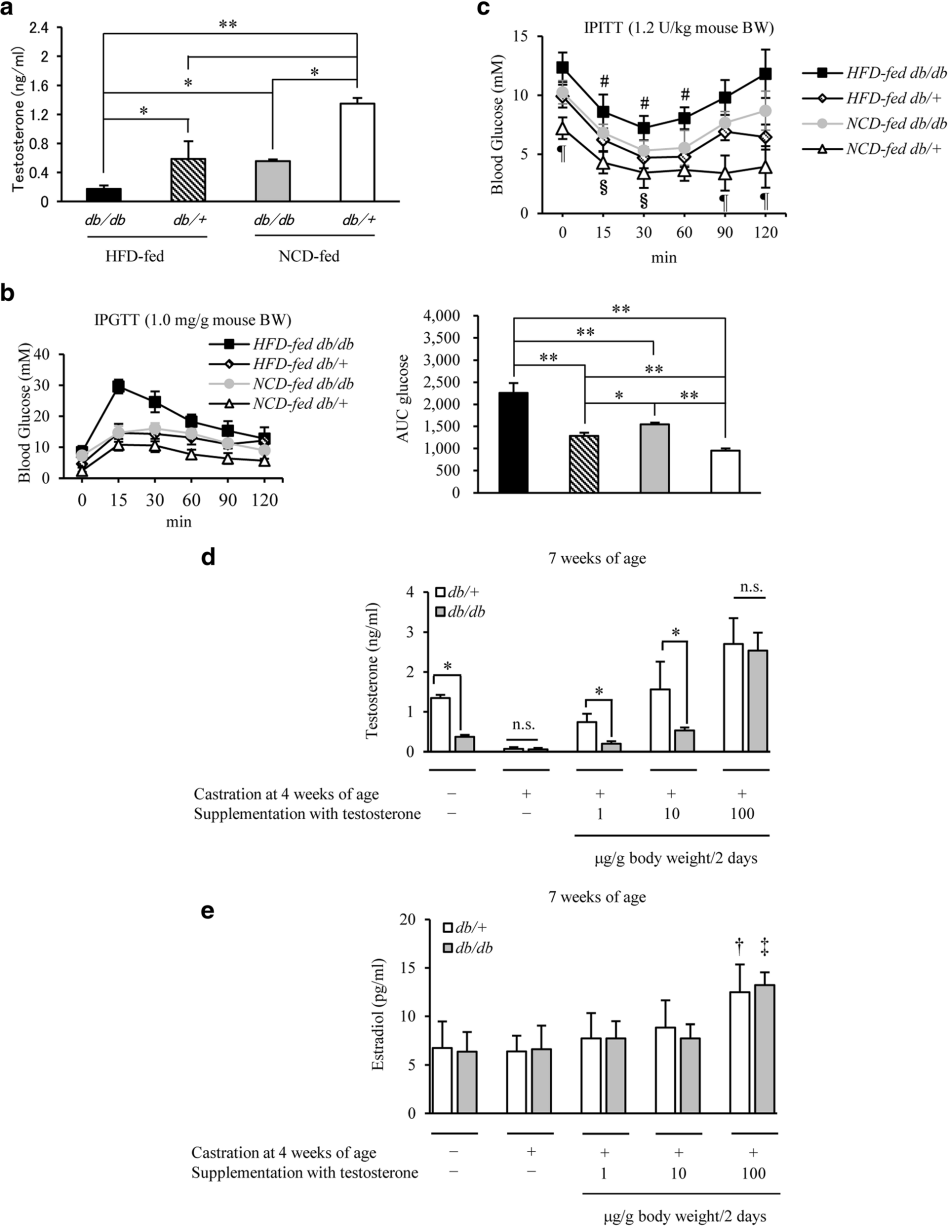
Induction of low levels of testosterone by leptin receptor KO or HFD. **a** Comparison of testosterone levels in NCD-fed *db/+* and *db/db* male mice, and HFD-fed *db/+* and *db/db* male mice, at 7 weeks of age (*n* = 14/group). Testosterone levels were the lowest in HFD-fed *db/db* mice, and increased in the order, NCD-fed *db/db* > NCD-fed *db/+* mice. **b** IpGTT analysis in 7-week-old mice on NCD or HFD in each group (*n* = 12–14/group). The area under the curve (AUC glucose) is shown in the right panel. Glucose homeostasis was exacerbated by HFD feeding. **c** IpITT analysis in 7-week-old mice in each group (*n* = 10–13/group) as indicated. **d** The dose of exogenous testosterone replacement and blood testosterone levels in castrated mice (*n* = 9–12/group). Exogenous testosterone was administered once every 2 days. Blood testosterone levels increased with an increase in the dose of testosterone replacement. **e** The dose of exogenous testosterone replacement and blood estradiol levels in castrated mice (*n* = 9–12/group). Blood estradiol levels did not significantly increase until the dose of exogenous testosterone replacement was elevated to 100 μg/g body weight/2 days. Results are expressed as means ± s.e.m. *P < 0.05, **P < 0.01 between the indicated groups. ^#^*P* < 0.01, ^¶^*P* < 0.01 versus the other group of mice at each time, 2-way ANOVA. ^§^*P* < 0.01 versus the other group of mice on NCD at each time, 2-way ANOVA. ^†^*P* < 0.05 versus another group of *db/+* mice. ^‡^*P* < 0.01 versus another group of *db/db* mice. n.s., no significant

A significant decrease in blood testosterone levels was observed in the order: HFD-fed *db/db* > NCD-fed *db/db* > NCD-fed *db/+* mice (Fig. 1a), which was consistent with the order of glucose intolerance observed in response to ipGTT and assessed by measurement of glucose area under curve (AUC) (Fig. 1b). Similar findings were also observed for ipITT analysis (Fig. 1c). Testosterone is known to be converted to estrogen by aromatase (aromatization); therefore, whether the effect of endogenous testosterone was direct or through estrogen remained unclear. Thus, 4-week-old *db/db* mice were castrated and treated with testosterone at various concentrations (1, 10 and 100 μg/g body weight/2 days) for 2 weeks from 5 weeks of age, and blood testosterone and estradiol levels were measured to establish the dose for testosterone replacement. Blood testosterone levels increased with an increase in the dose of testosterone (Fig. 1d). No significant increase in blood estradiol levels was observed at exogenous testosterone doses up to 10 μg/g body weight/2 days; however, a significant increase was noted at 100 μg/g body weight/2 days (Fig. 1e). Thus, the dose for testosterone replacement was set at 10 μg/g body weight/2 days.

### Impact of testosterone replacement on fuel homeostasis in castrated mice

**Fig. 2:**
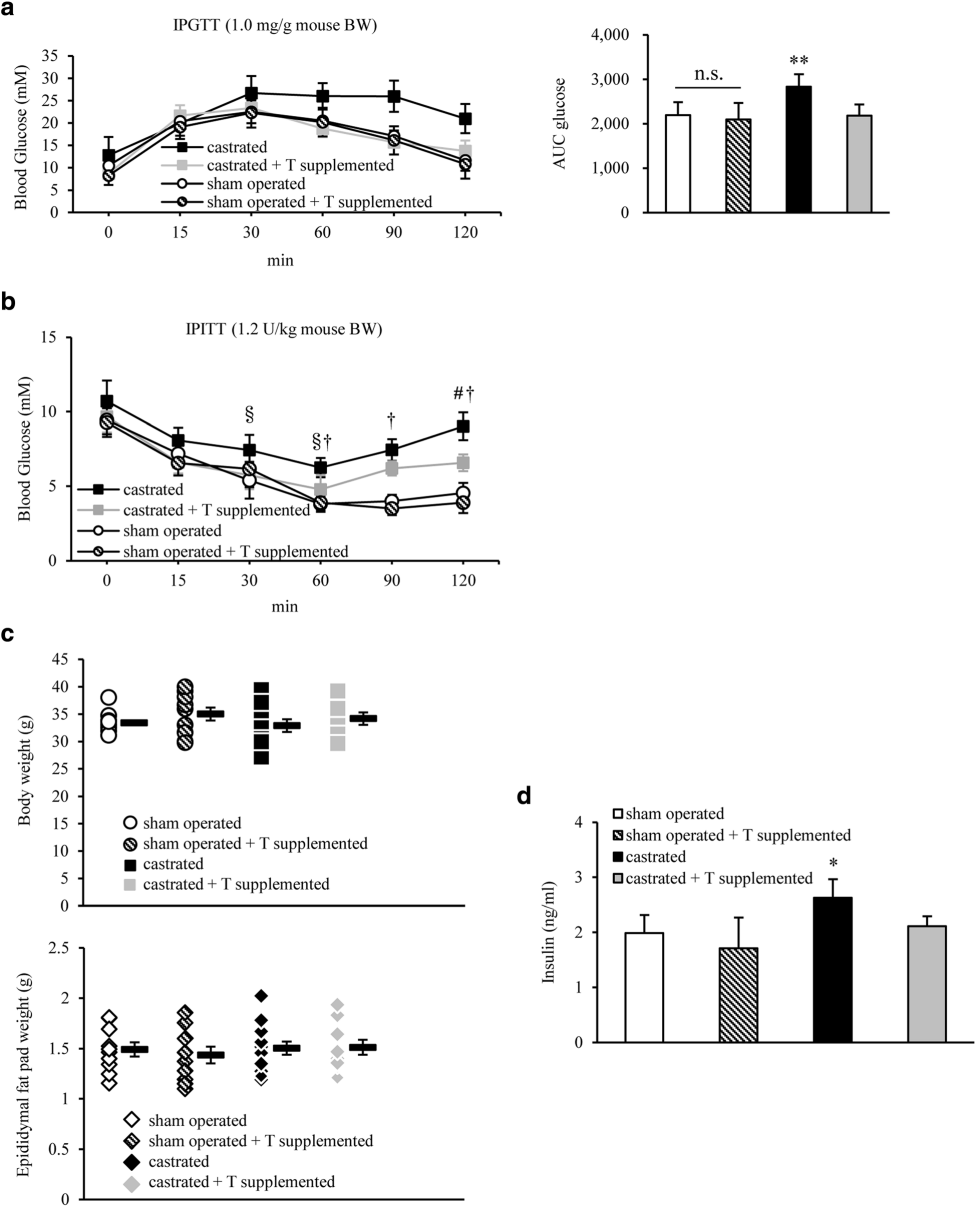
Glucose homeostasis in 7-week-old *db/db* mice was exacerbated by castration at 4 weeks of age, but was ameliorated by supplementation with testosterone. **a** IpGTT analysis in *db/db* male mice treated with castration, castration and testosterone (T) supplementation, or sham operation (*n* = 9–12/group). The area under the curve (AUC glucose) is shown in the right panel. T replacement significantly alleviated the exacerbation of glucose tolerance in castrated *db/db* mice. **b** IpITT analysis in 7-week-old mice in each group (*n* = 10–13/group) as indicated. Blood glucose levels 120 min after loading insulin were significantly higher in castrated than in sham operated mice, and showed a tendency to recover in the T replacement group. **c** Body weights of 7-week-old *db/db* mice in each group (sham operated, sham operated + T supplemented, castrated, or castrated + T supplemented mice; *n* = 10/group). The weight of epididymal fat from mice in each group is shown in the bottom panel as indicated. No significant difference was observed between the groups. **d** Fasting plasma insulin levels in each mouse (*n* = 12–14/group). Results are expressed as means ± s.e.m. *P < 0.05, **P < 0.01 versus the other groups of mice. ^§^*P* < 0.05, ^#^*P* < 0.01 versus castrated + T supplemented mice at each time. ^†^*P* < 0.01 versus sham operated or sham operated + T supplemented mice at each time, 2-way ANOVA. n.s., no significant

**Fig. 3:**
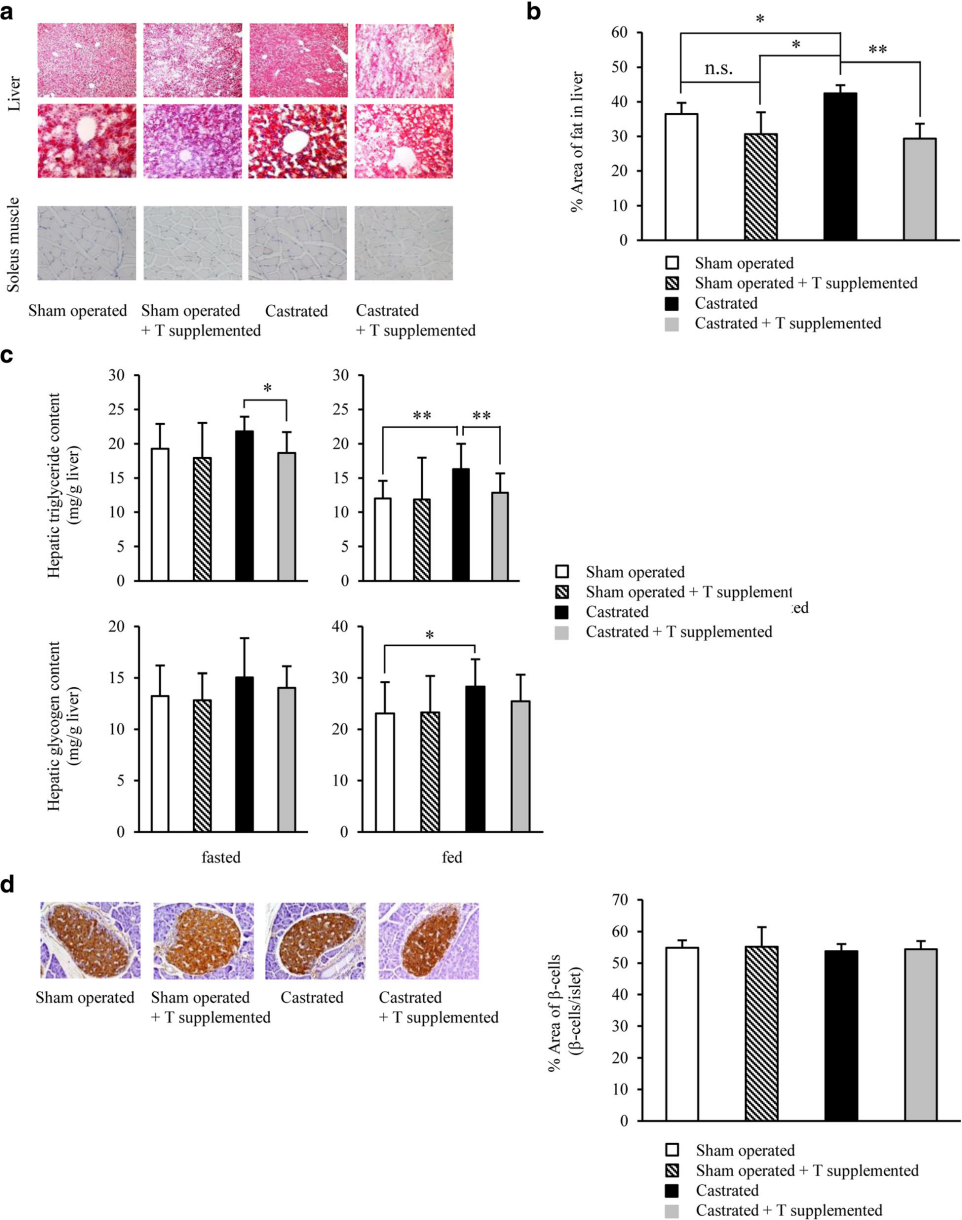
Decreased blood testosterone levels exacerbated fatty liver in *db/db* mice, and exogenous testosterone replacement mildly reduced this exacerbation. **a** Microscopic views of the liver [upper (× 40 magnification) and middle (× 200 magnification)] and soleus muscle [bottom (× 200 magnification)] from 7-week-old *db/db* mice in each group [sham operated, sham operated + testosterone (T) supplemented, castrated, or castrated + T supplemented mice; *n* = 9–12/group]. Hepatic steatosis was most exacerbated in castrated mice and reduced with T supplementation as shown by Oil Red O staining. **b** Oil Red O-positive area. **c** Hepatic TG (upper) and glycogen (bottom) content in fasted and ad libitum-fed (*n* = 11–13/group). **d** Histological analyses of the pancreas (*n* = 10–12/group). Representative sections stained with anti-insulin antibodies [left (× 100 magnification)]. The proportion of β cells was calculated relative to islets (right). Results are expressed as means ± s.e.m. *P < 0.05, **P < 0.01 between the indicated groups, 2-way ANOVA. n.s., no significant

IpGTT testing of the 4 HFD-fed groups—sham-operated, sham-operated+testosterone-supplemented, castrated, and castrated+testosterone-supplemented groups—at 7 weeks of age revealed that glucose tolerance was significantly lower in the castrated group than in the sham group, and also that testosterone replacement significantly reversed the exacerbation of glucose tolerance in castrated mice (glucose AUC: 2194 ± 295 in the sham group, 2101 ± 371 in the sham+testosterone-supplemented group, 2839 ± 280 in the castrated group, and 2182 ± 252 in the castrated+testosterone replacement group; *P* < 0.01) (Fig. 2a). IpITT testing also showed that insulin sensitivity was lower in the castrated group than in the sham group, and tended to recover in the testosterone replacement group (Fig. 2b). Therefore, glucose intolerance mainly associated with insulin resistance was attributed to a decrease in endogenous testosterone levels in castrated mice. No significant differences were observed in the body weight or epididymal fat weight among these groups at 7 weeks of age (Fig. 2c). In addition, fasting blood insulin levels were slightly higher in the castrated group, and were significantly decreased upon testosterone replacement (Fig. 2d). Fat accumulation in and the TG content of liver and muscle were also evaluated. The percentage area of fat was higher in the castrated group, and was significantly lower in the testosterone replacement group (% area of fat: 36.5 ± 3.2 in the sham group, 30.7 ± 6.3 in the sham+testosterone replacement group, 42.4 ± 2.4 in the castrated group, and 29.4 ± 4.3 in the testosterone replacement group; sham vs. castrated group, *P* < 0.05; castrated vs. testosterone replacement group, *P* < 0.01) (Fig. 3a and b). The testosterone replacement group also lowered liver TG content (Fasting state: 20.9 ± 6.9 mg/g liver in the sham group, 22.5 ± 4.6 mg/g liver in the castrated group, and 15.8 ± 1.9 mg/g liver in the castrated+testosterone replacement group; castrated vs. testosterone replacement group, *P* < 0.05; Fed state: 32.1 ± 10.6 mg/g liver in the sham group, 52.0 ± 13.3 mg/g liver in the castrated group, and 37.7 ± 11.1 mg/g liver in the castrated+testosterone replacement group; sham vs. castrated group, *P* < 0.01; castrated vs. testosterone replacement group, *P* < 0.01) (Fig. 3c). No significant differences were observed in these parameters or in the weight of the soleus muscle at 7 weeks of age (data not shown). Insulin resistance in obesity was previously shown to manifest as a result of impaired suppression of hepatic glycogen synthesis and glucose output. Therefore, the hepatic glycogen contents were assessed, demonstrating no significant difference between the groups after 16 h of fasting. However, they were significantly higher in the castrated mice than in the sham-operated mice in a fed state, and significantly reduced upon testosterone replacement (Fig. 3c). Pancreatic β cells were homogenously swollen in all groups, but no collapse (shrinkage of β cells) occurred, which indicated that a low testosterone level was unlikely to directly destroy pancreatic β cells (Fig. 3d).

### Impact of testosterone replacement on castration-induced insulin resistance in the liver

**Fig. 4:**
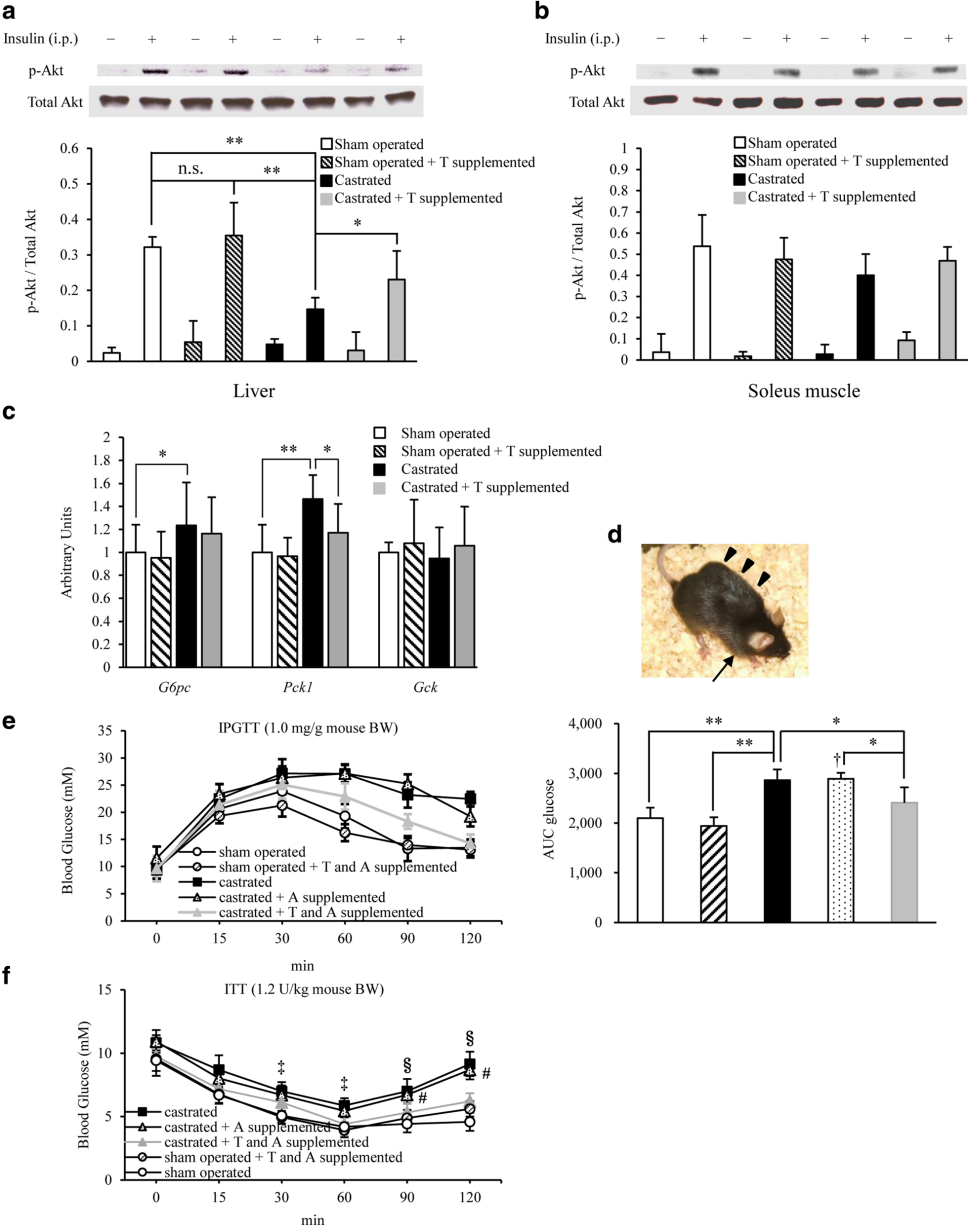
Low testosterone-induced suppression of hepatic insulin signaling played a certain role in the development of insulin resistance in castrated *db/db* mice. **a** Representative immunoblots (top) and the ratio (bottom) of hepatic phosphorylated Akt (p-Akt) versus total Akt in sham operated, sham operated + testosterone (T) supplemented, castrated, or castrated + T supplemented mice (7 weeks of age) treated from 4 to 7 weeks of age (*n* = 10–13/group). **b** The ratio of muscular p-Akt versus total Akt, as assessed by immunoblotting, in the mice (7 weeks of age) in each group (*n* = 8–11/group). **c** Hepatic mRNA levels of *G6pc*, *Pck1*, and *Gck* as determined by quantitative real-time PCR (qRT-PCR) at 7 weeks of age (16 h-fasted) (*n* = 10–13/group). **d** A photograph of a mouse receiving the continuous anastrozole infusion from an osmotic pump (arrowhead). In addition, a T pellet was implanted under the cervical skin (arrow). **e** IpGTT analysis in 7-week-old *db/db* mice fed NCD in each group (*n* = 8–10/group) as indicated. The area under the curve (AUC glucose) is shown in the right panel. Glucose homeostasis was exacerbated by castration and ameliorated upon T supplementation. **f** IpITT analysis in 7-week-old mice in each group (*n* = 8–9/group) as indicated. The blood glucose levels 120 min after insulin loading were significantly higher in castrated than in sham operated mice, and were significantly lower in the T replacement after castration group than in the castrated group. Results are expressed as means ± s.e.m. *P < 0.05, **P < 0.01 between the indicated groups. ^†^*P* < 0.01 versus sham operated or sham operated + T and A supplemented mice. ^‡^*P* < 0.05 versus sham operated mice at each time, 2-way ANOVA. ^§^*P* < 0.01, ^#^*P* < 0.01 versus castrated + T and A supplemented, sham operated + T and A supplemented, or sham operated mice, respectively, at each time, 2-way ANOVA

Insulin-inducing Akt signals were investigated in the liver and soleus muscle by immunoblotting. Akt phosphorylation was significantly decreased in the liver of the castrated group, and recovered upon testosterone replacement (Fig. 4a). However, no significant difference was observed in Akt signals in the soleus muscle (Fig. 4b). A comparison of the relative *G6pc* and *Pck1* mRNA/*Gapdh* expression levels in the liver revealed that these levels were enhanced in the castrated group, and then the *Pck1* levels were mildly but significantly reduced upon testosterone replacement (Fig. 4c).

Based on the above findings, low blood testosterone levels appear to somewhat inhibit insulin signaling in the liver during the relatively young period in *db/db* mice.

Testosterone is almost not affected by circadian rhythms. To reduce subcutaneous testosterone injection-induced variations in blood testosterone levels (i.e., to prepare a steady state), a testosterone pellet was implanted under the cervical skin in castrated mice at 4 weeks of age (a placebo pellet was implanted in the control mice). In addition, to completely block the influence of aromatization, the aromatase inhibitor, anastrozole, was simultaneously and continuously infused using an osmotic pump (Fig. 4d). We found that aromatization was completely blocked by anastrozole administration (Additional file 1: Figure S1). When glucose metabolism were evaluated in 7-week-old mice, the results obtained were similar to those in animals treated with a subcutaneous testosterone injection alone (Fig. 4e and f), which suggests that the recovery of glucose metabolism with testosterone replacement involved at least the direct action of testosterone.

### Aging- and dietary content-induced changes in blood testosterone levels and the influence of female mice in the reproductive period on blood testosterone levels in male mice

**Fig. 5:**
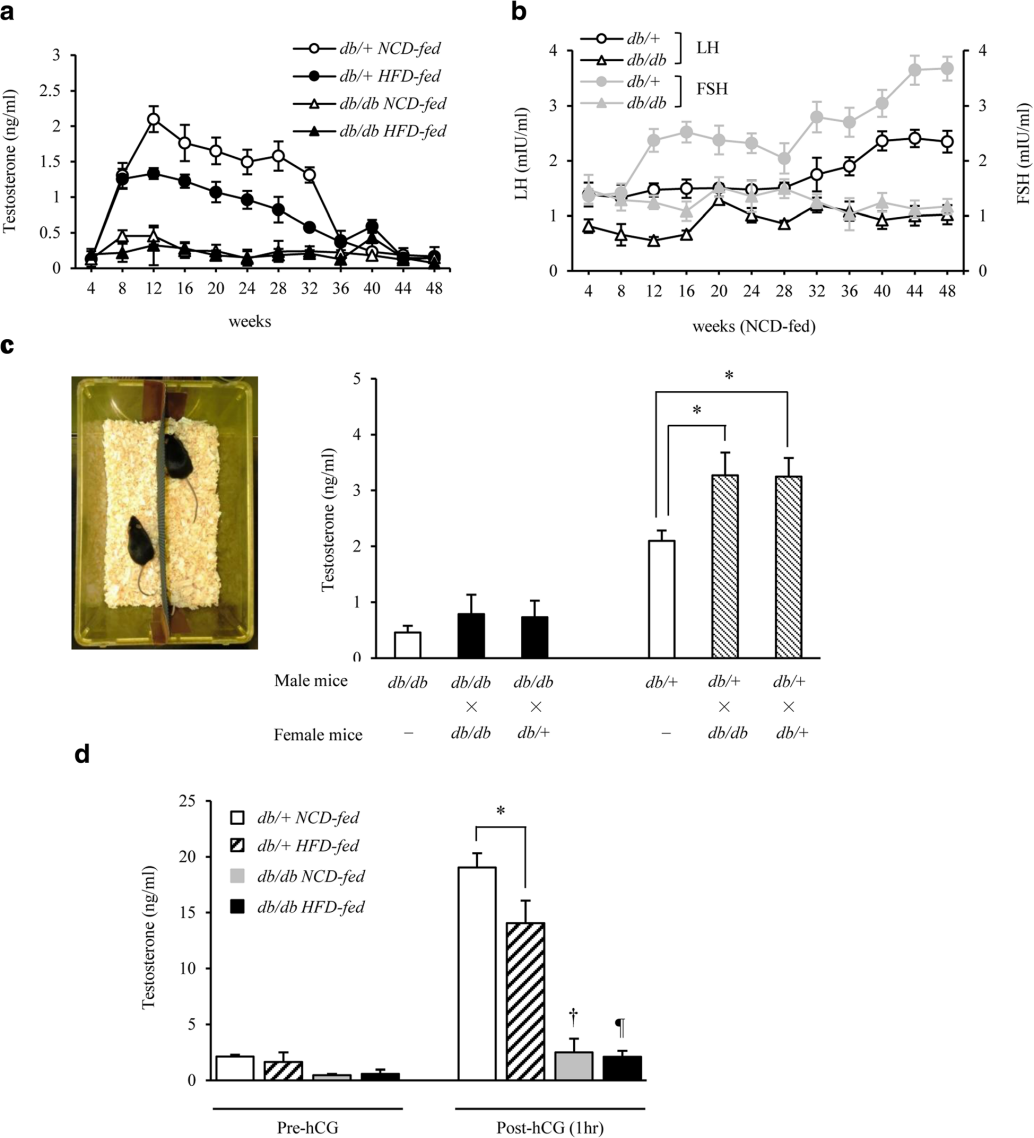
Blood testosterone levels in male *db/db* mice were low throughout life from the juvenile period, and their reactions to female mice and hCG were weak. **a** Changes in blood testosterone levels from 4 to 48 weeks of age in each group are shown (*n* = 9–11/group). Blood testosterone levels rapidly decreased in NCD-fed male *db/+* mice at 36 weeks of age and older. These levels were lower in male *db/db* mice than in male *db/+* mice throughout life, and were also lower in male *db/+* mice in the HFD- than in NCD-fed group. **b** The changes in gonadotropin levels from 4 to 48 weeks of age in each group are shown (*n* = 9–11/group). The blood LH and FSH levels increased in male *db/+* mice after 32 weeks of age. In contrast, these levels were low in male *db/db* mice throughout life. **c** A pair of male and female mice were kept in the same cage for 2 weeks (from 10 to 12 weeks of age) in each group (left picture), and the blood testosterone levels were measured in male mice after 2 weeks (right) [*n* = 8–14 (7–8 males and 0–7 females)/group]. The presence of a female significantly increased blood testosterone levels in male *db/+* mice, whereas no significant change was noted in male *db/db* mice. **d** Increases in blood testosterone levels after hCG loading (0.5 IU/g body weight) in 12-week-old male *db/+* and *db/db* mice (*n* = 10/group). hCG increased blood testosterone levels by nearly 10 times in male *db/+* mice, but only by approximately 2 times in male *db/db* mice. Results are expressed as means ± s.e.m. *P < 0.01 between the indicated groups. ^†^*P* < 0.01, ^¶^*P* < 0.01 versus pre-hCG in *db/db* mice on each diet

As shown in Fig. 5a, changes in blood testosterone levels with aging were compared between male *db/db* and *db/+* mice. Blood testosterone levels decreased with aging after reaching a peak during the reproductive period in male *db/+* mice. In contrast, these levels were already low from the juvenile period in male *db/db* mice, and remained low even during the reproductive period. Regarding the effect of the diet (NCD and HFD) on male *db/+* mice, blood testosterone levels were lower in the HFD- than in the NCD-fed group throughout the observation period (Fig. 5a). The levels of simultaneously measured LH and FSH gradually increased in male *db/+* mice at 32 weeks of age and older, but remained low in male *db/db* mice throughout the observation period from 4 weeks of age to the elderly period (Fig. 5b). In addition, the testis weight was lower in *db/db* mice than in age-matched *db/+* mice (Table 1).

**Table 1:** Mean body and testes weights of *db/+* and *db/db* mice at 12 and 40 weeks of age

	*db/+* mice	*db/db* mice	*P* value
Body weight (g)			
12 wk	28.42 ± 0.82	48.14 ± 0.61	0.002
40 wk	35.06 ± 0.67	56.86 ± 0.91	0.004
Testes weight (g)			
12 wk	0.194 ± 0.009	0.131 ± 0.003	0.015
40 wk	0.186 ± 0.009	0.148 ± 0.011	0.031
Ratio of testes / body weight (%)			
12 wk	0.68 ± 0.02	0.30 ± 0.05	0.001
40 wk	0.53 ± 0.06	0.26 ± 0.08	0.001

Data are expressed as means ± SE, unless stated otherwise. Significance was considered as *P* < 0.05, calculated by an independent two-sample *t* test

To investigate the influence of mating on blood testosterone levels, on the assumption that mating elevates these levels, male and female mice were kept in the same cage for 2 weeks from 10 weeks of age (Fig. 5c). Blood testosterone levels significantly increased in 12-week-old male *db/+* mice regardless of whether the female mice in the same cage were *db/+* or *db/db*. In contrast, no significant increase was observed in blood testosterone levels in 12-week-old *db/db* mice in the presence of female mice of either genotype. hCG is known to stimulate the production of testosterone [39]. The hCG load test revealed that the increase in testosterone levels was significantly lower in male *db/+* HFD-fed mice than in *db/+* NCD-fed mice at 12 weeks of age. In contrast, although the hCG stimulating, testosterone levels were low in male *db/db* mice (Fig. 5d).

## Discussion

In an obesity model, *db/db* mice, a decrease in blood testosterone levels significantly aggravated glucose intolerance even at a relatively young age. Previous reports have suggested that AR acts to reinforce the action of leptin [25–27]; however, our findings, obtained with the leptin receptor knockout *db/db* mice, indicate the presence of a pathway not mediated by leptin signaling in the mechanism by which testosterone inhibited the exacerbation of glucose tolerance. To the best of our knowledge, no report has yet confirmed that fatty liver generally develops in men with hypogonadism, e.g., men with Klinefelter syndrome, Prader-Willi syndrome or Kallmann syndrome, or after castration. In this context, the actions of leptin and testosterone may complement each other, while their simultaneous reduction has been attributed to the aggravation of glucose tolerance observed in our model. Based on the results obtained from aromatization, we set the dose of exogenous testosterone replacement at 10 μg/g body weight/2 days (Fig. 1d and e). Variation of the blood testosterone level was marked in *db/+* mice supplemented with 10 μg/g body weight/2 days of testosterone, but the level was significantly higher than that in *db/db* mice, whereas no difference was noted in the blood estradiol level. Since exogenous supplementation with 10 μg/g body weight/2 days of testosterone did not reach the aromatization-inducing threshold in this mouse strain (both *db/+* and *db/db*), it was considered that sufficient conversion to estradiol by aromatization did not occur unless the dose of exogenous testosterone was increased to 100 μg/g body weight/2 days. Thus, we performed an additional experiment with 75 μg/g body weight/2 days of testosterone, and observed that the blood estradiol level was significantly higher in *db/+* than *db/db* mice (*db/+* mice vs. *db/db* mice; 10.5 vs. 7.6 pg/mL) (data graph not shown). However, a small amount of estradiol may still have been produced by aromatization because of variations in blood testosterone levels and the influence of glucose and lipid metabolism. To remove this possibility, a constant blood testosterone level was established by implanting a testosterone pellet under the cervical skin. Combined with the administration of anastrozole, the influence of aromatization was completely blocked and similar results were obtained (Fig. 4e and f, and Additional file 1: Figure S1). These findings indicate that testosterone directly alleviated the exacerbation of glucose tolerance, but not through leptin signaling. Although no significant difference was observed in the β cell rate relative to islets (Fig. 3d), testosterone replacement significantly improved insulin sensitivity (Fig. 2b) and decreased fasting insulin levels (Fig. 2d) accompanying mildly reduced hepatic fat accumulation and TG content (Fig. 3a–c) in castrated mice, suggesting that testosterone directly or indirectly decreases intrahepatic fat or may influence insulin-degrading enzyme (IDE). In each group, the power calculation values were low for fasting insulin levels, hepatic triglyceride contents, hepatic glycogen contents, and real-time PCR results (Additional file 2: Figure S2). However, they showed a tendency for significant differences, which could be simply explained by the small sample size.

We initially expected the recovery of insulin sensitivity in muscle to play an important role in glucose tolerance in this experimental system because testosterone increases muscle mass. However, no significant differences were observed in the fat content of, or Akt signals in, the soleus muscle between sham, castrated and testosterone-treated mice. Because no significant difference was noted in the soleus muscle weight among the groups at 7 weeks of age (data not shown), a 2-week testosterone replacement may have been too short to recover insulin sensitivity in muscle, and prolonging the replacement period may have led to different results.

Regarding the influence of aging, blood testosterone levels reached a peak in the reproductive period and then slowly decreased until 1 year of age in *db/+* mice (Fig. 5a), a pattern that is similar to that in healthy men. In contrast, blood testosterone levels in *db/db* mice were low throughout the period from 4 weeks to 1 year of age (Fig. 5a). Based on a comparison of the findings reported by Garris DR et al. [40], the decreased LH levels characteristic of *db/db* mice may have influenced these levels (Fig. 5b). This difference in gonadotropin levels was also implicated in the differences observed in the testis weight (Table 1). The decreased secretion of gonadotropin-releasing hormone (GnRH) from the hypothalamus may also have been attributed to the decreased gonadotropin level measured in *db/db* mice; however, this needs to be investigated in more detail in future studies. Leptin gene knockout mice (*ob/ob* mice) are infertile. However, as reported by Chehab et al., female mice show restored fertility after receiving recombinant leptin [41]. Moreover, as reported by Mounzih et al., male *ob/ob* mice show restored fertility with estrus induction and testis weight gains as blood gonadotropins, especially LH, increase after administration of recombinant leptin [42].

In addition, as demonstrated by Wabitsch et al., young male patients with anorexia nervosa had lower gonadotropin (LH and FSH) and testosterone levels with decreasing blood leptin levels and, surprisingly, these parameters recovered along with weight gains (increases in body fat) [43].

The lower testosterone and gonadotropin levels with aging in the leptin receptor knockout mice (*db/db* mice) were comparable to those of *ob/ob* mice. However, the parameters were not recovered by the administration of recombinant leptin in *db/db* mice, unlike in *ob/ob* mice. Furthermore, although the infertility of *ob/ob* mice can be clearly explained by defects in the hypothalamic-pituitary axis, blood gonadotropin levels are elevated in castrated male *ob/ob* mice [44]. This finding suggests the existence of partial negative feedback in the hypothalamic-pituitary-gonadal axis. *db/db* mice showed non-significant negative feedback (slight elevations of gonadotropins) after castration, which disappeared after testosterone supplementation (data not shown). This result also has important implications in humans. Specifically, gonadal functions may decline after unnecessary testosterone supplementation in young men with low gonadotropin and testosterone levels due to decreased leptin.

Regarding dietary content, markedly lower blood testosterone levels were observed in male HFD-fed *db/+* mice than in NCD-fed mice (Fig. 5a). Although we did not investigate the dietary components in detail, obesity or the exacerbation of glucose and lipid metabolism induced low blood testosterone levels, which further promoted the aggravation of metabolic parameters, resulting in a vicious cycle. Decreased leptin signals may also have been strongly associated with this vicious cycle; however, further studies are needed to elucidate this mechanism in more detail.

When male and female mice were kept in the same cage during the reproductive period, blood testosterone levels were low in male *db/db* mice, possibly because of decreased LH levels (Fig. 5b and c). In addition, the hCG load test revealed that blood testosterone levels were nearly 10 times higher after hCG loading in *db/+* mice, while this increase was significantly lower in *db/db* mice (Fig. 5d). These findings indicate either that in addition to pituitary hypogonadism, functional Leydig cells are almost absent in *db/db* mice, or that the presence of leptin resistance or phenotypic obesity in males may suppress increases in blood testosterone levels. These findings support the hypothesis that testosterone inhibits the development and progression of lifestyle-related diseases, mainly diabetes [8, 17]. However, we did not investigate the influence of androgens produced by the adrenal gland. Moreover, elevation of the hematocrit value [45] and cardiovascular risk [46] resulting from an increase in the blood testosterone level is of concern, but the increased risk of prostate cancer remains controversial [3, 47–49]. Furthermore, whether changes in blood testosterone levels are the cause or result of various diseases remains to be clarified. Because systemic disease may decrease testosterone levels, a reverse causal relationship should also be considered [50, 51]. Therefore, testosterone should not be administered to middle-aged to elderly men that do not have decreased testosterone levels (those that do not meet the diagnostic criteria of a general androgen deficiency). However, testosterone replacement was shown to be beneficial for men with decreased testosterone levels in several studies [52–54]. Therefore, the advantages and problems associated with testosterone replacement therapy need to be elucidated in more detail in future studies.

## Conclusion

Our results strongly suggest that there are two pathways whereby testosterone affects glycometabolic functions: an indirect pathway via leptin signaling and a direct pathway that acts in a leptin-independent manner.

## Additional files

Additional file 1: **Figure S1.** Aromatase inhibitor anastrozole blocks conversion of exogenous testosterone to estradiol (aromatization). The continuous anastrozole infusion rate was adjusted to 0, 20, or 200 μg/day (*n* = 8–10/group). A significant increase in blood estradiol levels was observed at an exogenous testosterone dose of 100 μg/g body weight/2 days, which were significantly decreased upon anastrozole infusion (20 or 200 μg/day) in castrated *db/db* mice. Results are expressed as means ± s.e.m. *P < 0.01 versus the other group of castrated *db/db* mice on the NCD. (ODP 17 kb)

Additional file 2: **Figure S2.** Power analysis of each experiment. After each experiment, we performed a post-hoc power analysis to assess the power of the experiment. Results are expressed as 1-β error probabilities. (ODP 20 kb)

## Abbreviations

AR: Androgen receptor
AUC: Area under curve
DAB: Diaminobenzidine
DHT: Dihydrotestosterone
ECL: Enhanced chemiluminescence
ELISA: Enzyme-linked immunosorbent assay
FSH: Follicle stimulating hormone
GnRH: Gonadotropin-releasing hormone
hCG: Human chorionic gonadotropin
HDL: High-density lipoprotein
HFD: High-fat diet
IDE: Insulin-degrading enzyme
ipGTT: Intraperitoneal glucose tolerance test
ipITT: Intraperitoneal insulin tolerance test
KO: Knockout
LH: Luteinizing hormone
MetS: Metabolic syndrome
NCD: Normal chow diet
PPARγ: Peroxisome proliferator-activated receptor gamma
QOL: Quality of life
SHR: Spontaneously hypertensive rat
STAT3: Signal transducer and activator of transcription 3
TG: Triglyceride
